# An Opportunistic Routing Mechanism Combined with Long-Term and Short-Term Metrics for WMN

**DOI:** 10.1155/2014/432123

**Published:** 2014-08-26

**Authors:** Weifeng Sun, Haotian Wang, Xianglan Piao, Tie Qiu

**Affiliations:** Software School, Dalian University of Technology, Dalian 116620, China

## Abstract

WMN (wireless mesh network) is a useful wireless multihop network with tremendous research value. The routing strategy decides the performance of network and the quality of transmission. A good routing algorithm will use the whole bandwidth of network and assure the quality of service of traffic. Since the routing metric ETX (expected transmission count) does not assure good quality of wireless links, to improve the routing performance, an opportunistic routing mechanism combined with long-term and short-term metrics for WMN based on OLSR (optimized link state routing) and ETX is proposed in this paper. This mechanism always chooses the highest throughput links to improve the performance of routing over WMN and then reduces the energy consumption of mesh routers. The simulations and analyses show that the opportunistic routing mechanism is better than the mechanism with the metric of ETX.

## 1. Introduction

Wireless mesh network based on IEEE 802.11 is a wireless multihop network which is easy to deploy, is of low cost, and has wide coverage. WMN is an efficient extension of wired network. It overcomes the limitation of the harsh geographical conditions and provides high-speed transmission. WMN is also the suitable access solution of last mile. However, compared with wired network, the channel of wireless networks cannot provide a guaranteed link, and there also exists asymmetric, immediate loss and transmission error (bit error) in wireless networks [1]. From the research in [2], data packet loss is not caused only by distance and signal attenuation; SNR (Signal-to-Noise Ratio, S/N) and multipath fading with interference are also the important reasons which lead to packet loss. In wireless networks, SNR and BER (Bit Error Ratio) should be taken account of the information of neighbor nodes; thus, the information is relatively unreliable. Traditional link-state and distance vector based routing protocols such as OSPF (Open Shortest Path First Interior Gateway Protocol) [3] and RIP (Routing Information Protocol) [4] do not work very well. According to traditional routing protocols, packet loss which is caused by physical layer will react to the transport layer and lead to the unavailability of users' services.

In [5], Ahmeda and Esseid claimed that each individual routing metric considers some features and it is difficult to satisfy all the requirements of WMNs by using a single metric. So we consider combining two different metrics to avoid the disadvantage of single metric.

**Figure 1:**
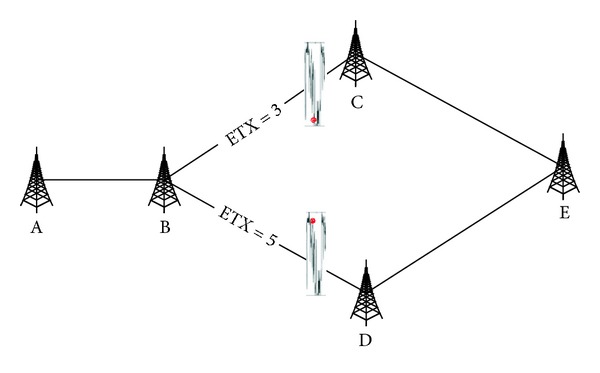
The state of links in data transmission.

Figure 1 is a simple example of the problem of ETX. The data flow starts from node A to node E, and the ETX of links B → C and B → D are 3 and 5, respectively. According to traditional mechanism with the routing metric of ETX, node B will choose node C as the next hop to transmit. If the link of B→ C has a lower probability of successful transmission, while the probability of successful delivery over BD is higher at the same time, the link of B → D should be chosen to achieve better performance of transmission.

In this paper, we study the problem of original ETX used in WMN. We consider a wireless mesh network deployed in a simple topology which has three source destination pairs of data which start simultaneously. Two important parameters that are related to the problem are data transmission rate and probe interval of ETX. Particularly, we are interested in the following three specific questions.

- What is the optimized probe interval of ETX that leads to maximum throughput of WMN?
- Does the data rate of transmission of WMN effect the optimization of probe interval of ETX?
- Could another metric be added in the original ETX to overcome the shortcoming of original ETX?

Through the cross-layer process between MAC, routing, and application layers, a quality aware routing with opportunity based on ETX is proposed in this paper. This mechanism which is under the assurance of QoS with long-term (longer probe interval for calculating the metric) routing metric (ETX, expected transmission count) concludes a candidate forwarding link set. Then, according to short-term (shorter probe interval for calculating the metric) routing metric (RTT, Round-Trip Time), the link set will be checked and the candidate link with the best wireless link condition will be chosen by this mechanism to transmit packets. Through the combination of long-term and short-term routing metrics, this opportunistic routing mechanism not only chooses the link with smaller ETX to assure the high probability of transmission success, but also guarantees that the transmission over each hop has higher throughput and improves the efficiency of data transmission.

The contribution of our work includes the following aspects:

- analyses of the disadvantage of ETX over WMN with different data transmission rates and probe intervals of ETX through experiments of simulation;
- we propose an opportunistic routing mechanism combined with long-term and short-term metrics based on OLSR and ETX to overcome the disadvantage and improve the routing performance;
- analyses of theory and implementation to prove the advantage of the opportunistic routing mechanism;
- simulation results evaluate the performance of the opportunistic routing mechanism, and it performs better than ETX.

The rest of paper is organized as follows: Section 2 introduces the background of ETX, formulates the network problems under study, and reviews related work. The opportunistic routing mechanism is described in Section 3.  Section 4 gives the analyses and performance evaluation of the mechanism. Finally, Section 5 summarizes the paper and our future work.

## 2. Related Work

In this section, we introduce the background of ETX and formulate the network model under study and review related work.

### 2.1. Background of ETX

The routing metric of WMN includes hop count [6], RTT [7], Per-Hop Packet Pair Delay (PktPair) [8], ETX, expected transmission time (ETT) [9], and Weighted Cumulative Expected Transmission Time (WCETT) [10]. The selected path by routing algorithm may be the one of the farthest, weakest signal and the least energy with the routing metric of hop count. In particular, in dense scenario, the routing algorithm with minimum hop count does not work efficiently and the chosen path has weak signal and low bandwidth and it brings more interference, so a new routing metric in wireless multihop network is needed. According to the research in [11], the results in the test-bed of Roofnet show that the probability of one packet's successful transmission cannot be predicted accurately. So ETX with statistical function according to the Allen variance was designed. ETX calculates the expected transmission count of a packet over a link. ETT, WCETT, and some others are all the routing metrics based on ETX. From the results of tests with variety of routing metrics in [12], the metric of hop count reflects the actual topology state better and achieves better performance ad hoc, while, for WMN which has relatively fixed nodes, ETX performs better.

In ETX, a mesh router delivers the probe packets to its neighbor nodes every 1 second. Through the statistic of successful forwarding count *d*_*f*_ and successful receiving of ACK (acknowledgment) count *d*_*r*_, EXT calculates the expected count of failure forwarding and receiving *P*_*f*_ and *P*_*r*_. So the probability of failure transmission is *P* = 1 − (1 − *P*_*f*_)∗(1 − *P*_*r*_) and the probability of successful transmission after *k* times' retransmission is *s*(*k*) = *P* ^*k*−1^∗(1 − *P*). By summing, the expected successful transmission count is

(1) $$\begin{array}{c} \text{ETX}=\overset{\infty }{\underset{k=1}{\sum }}k*s\left(k\right)=\frac{1}{1-P}=\frac{1}{\left(1-P_{f}\right)*\left(1-P_{r}\right)}. \end{array}$$

ETX calculates the expected transmission count through the statistical results in the latest 10 seconds and updates every 1 second. If the quality and transmission rate of link fluctuate wildly in a certain period of time, it will not work well for this method which predicts current transmission rate according to the historical data in wireless multihop networks.

### 2.2. Related Work

In this section, we briefly review existing work on the improvement of ETX in WMN.

It will encounter some problem when the metric of ETX is used over WMN in the practical scenario. Some new metrics based on ETX were proposed in several literatures. Through this kind of method, most of these new metrics could reflect network conditions more accurately. In [13], Koksal and Balakrishnan describe two new metrics, called modified expected number of transmissions (mETX) and effective number of transmissions (ENT) that work well under a wide variety of channel conditions. Empirical observations of a real-world wireless mesh network suggest that mETX and ENT could achieve a 50% reduction in the average packet loss rate compared with ETX.

In order to solve the problem that ETX performs poorly in the case of multiradio and multichannels, WCETT was proposed in [14] to extend ETX and make it support multichannels. Instead of the probe mechanism with single minimum rate, WCETT adopts the probe mechanism with multirates to predict the packet loss rate accurately.

In the implementation of ETX, it acquires the global information firstly and selects the path from the source point to the end point with minimum expected transmission count. This kind of method according to the expected transmission count (including retransmission count) considers the influence of packet loss rate and asymmetry of link in wireless network. Mogaibel et al. experimentally verified ETX's high-speed transmission rate, and the performance was improved most especially on single-channel WMN in [12].

Also, the metric of ETX was implemented over different multihop routing protocols like optimized link state routing (OSLR) protocol [15]. OLSR adopts a proactive, optimized link state scheme to spread topology information while keeping the message overhead low. The key idea is that link-state information is generated and flooded in the network only by selected nodes, called Multipoint Relays (MPRs). Any source-destination route is bidirectional and includes only MPRs as relay nodes. OLSR has been extensively used around the world for building low-cost community owned mesh networks and the metric of ETX has been added in OLSR protocol in several researches.

Based on ETX over OLSR, some modification of this protocol was proposed. This kind of method usually modifies the details of metric and makes the metric overcome the disadvantage of ETX over OLSR. In [16], Johnson and Hancke presented an experimental comparison of OLSR using the standard routing metric and ETX metric in a 7 by 7 grid of closely spaced Wi-Fi nodes. The results show that the ETX metric which has been extensively used in mesh networks around the world is fundamentally flawed when estimating optimal routes in real mesh networks. Houaidia et al. pointed out the shortcoming of the ETX metric for eventual optimizations towards a more efficient routing through using several real experiments [17]. And then they presented improvements of the ETX metric based on link availability for accurately finding high throughput paths in multihop wireless mesh networks. In [18], Pinheiro et al. proposed the OLSR-Fuzzy ETX Queue (OLSRFEQ) protocol to overcome the limitations of OLSR-ETX regarding queue availability and QoS and QoE assurance. OLSR-FEQ optimizes network and user-based parameters by coordinating queue availability, QoS, and fuzzy issues in the routing decision process as a way of allocating the best paths for multimedia applications.

On the other hand, ETX was also implemented over AODV [19], and modifications of ETX based on AODV are proposed in current research. In [20], Ni et al. proposed a modified solution in which they repeatedly broadcast RREQ (Route Request) packets. Simulation results show that their modified solution improves ETX in the initial route selection in both single flows and multiple flows cases.

In another work of ours, we try to implement a mechanism which changes the probe interval of ETXs probe packet to adapt different transmission rates of wireless mesh network. Different from previous methods, this mechanism does not modify the protocol itself but attaches a cross-layer module to dynamically change the value of factors in protocol according to network conditions.

In order to adjust to the variety of wireless networks and reflect link state more accurately, referencing the idea of ExOR in [21] an opportunistic routing mechanism combining long-term and short-term metrics is designed: every mesh router maintains a set of forwarding links and each candidate link in this set has the opportunity to be chosen. In this scheme, the metric of ETX plays the role of main factor in the set and every mesh router will change the set according to the values of ETX. This mechanism not only will choose the link with minimum ETX, but also avoids data transmission over the link with poor quality. Also, this mechanism will enlarge the scale of transmission and improve the performance of transmission under the assurance of users' QoS.

## 3. Network Model

To understand our proposed mechanism better, we provide an overview of the network model assumed in this section. We consider a wireless mesh network deployed on a simple topology which has three competing data flows. One of the flows needs relay nodes to transmit packets and there are two different ways to transmit. The other two flows are used to influence the routing of ETX. This wireless mesh network adopts the protocol of IEEE 802.11 and antenna type is omniantenna. The nodes in the network are fixed and density of network is also fixed; however, the transmission rate of every node is variable. And hop count of this network is from 1 to 3.

In the network model, we assume that the size *f* data packet transmitted over the wireless link is the same as the size of probe packet which is used to acquire the information of RTT. In our opportunistic outing mechanism, ETX_threshold_ is a threshold to filter candidate links and it should be optimized to achieve better outing performance. The notations used in this paper can be found in Notations.

## 4. Opportunistic Routing Mechanism

### 4.1. Disadvantage of ETX

We did simulation with a simple topology based on NS-2 [22] as follows: simple topology and random topology. Through the evaluation metrics of throughput, end-to-end delay, and packet delivery rate, we find the problem that the original ETX will not always perform well when the total data rate of WMN changes rapidly. That is because its fixed probe interval will not adapt to the change of network environment.

**Figure 2:**
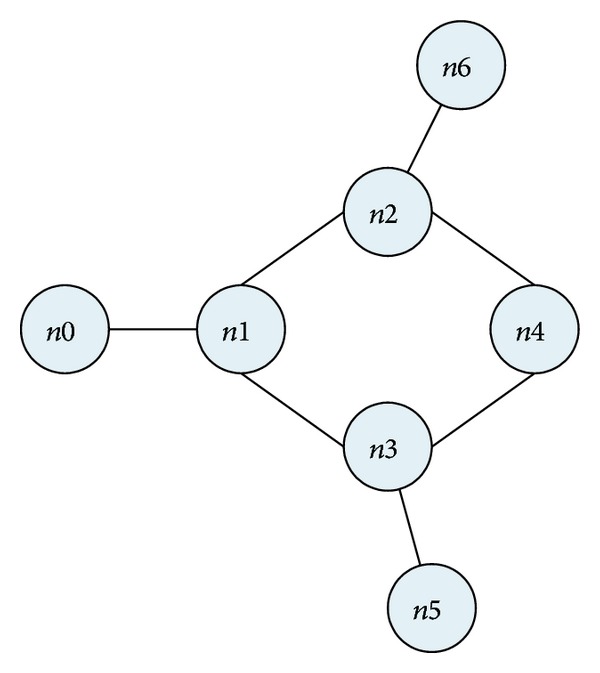
Topology of simulation.

To verify the weakness of the routing metric of ETX, we process the simulation as follows: Figure 2 is a simple topology that is used in our simulation studies. It consists of 7 nodes which are static in the 2000 m∗2000 m ground. The maximum transmission range of nodes is 250 m and the distance between any two neighboring nodes makes one node only communicate with its neighboring nodes directly. Node* n*0 transmits data packets to the destination node* n*4 and there are other two data flows:* n*6 →* n*2 and* n*5 →* n*3.

Our simulator is NS-2.34 and the routing protocol in the simulation is UM-OLSR [23] which is the extension of OLSR with ETX [24]. And the other settings of parameters in our simulation are shown in Table 1.

We vary the probe interval from 0.5 second to 4.0 (0.5, 1.0, 1.5,…, 4.0) seconds with different data transmission rates of the data flow* n*0 →* n*4 which is from 0.5 Mbps to 2.0 Mbps, and the data transmission rates of the other two data flows* n*6 →* n*2 and* n*5 →* n*3 are 0.3 Mbps and 1.0 Mbps, respectively. This simulation lasts 50 seconds and these data flows all start at 10 s.

We get the statistical results of throughput, delay, and packet delivery rate of node* n*4 during the simulation.

**Figure 3:**
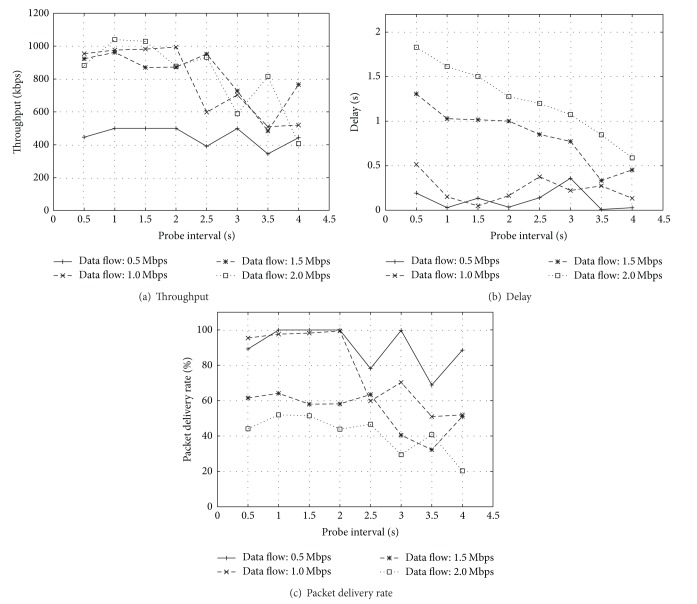
Performance with different probe intervals and data flow rate.

Figure 3(a) shows that the throughput will decrease with the increment of probe interval when the data rate is not very low (1.0, 1.5, and 2.0 Mbps). However, the throughput will not change a lot with the increment of probe interval when the data flow is low (0.5 Mbps). That is because the collision of packets on WMN is not very severe and the bandwidth of network is wide enough for data packets and probe packets. While the collision between data packets and probe packets will increase when the data rate is high, we could set the probe interval as a smaller value to achieve higher throughput. That is because the more frequently the probe packets are transmitted, the more quickly ETX mechanism could select the better path to transmit data packets. But we can see that there exist inflection points of probe interval which makes the throughput increase (3.5 seconds of probe interval with 2.0 Mbps of data rate). The reason of this phenomenon is that the advantage of reducing packet collisions recovers the disadvantages of switching to the better path slowly. However, to the higher rate of data flow, the longer probe interval will achieve lower end-to-end delay which could be seen in Figure 3(b). So the longer probe interval could be a better choice when the data rate is higher. And in Figure 3(c) we can see almost the same circumstance which is shown in Figure 3(a).

From the results of simulation, we can find that too long probe interval will make ETX perform poorly, while too short interval will cause the waste of bandwidth and lead to longer end-to-end delay. And there exists an optimum interval which will achieve the best performance of ETX, while this interval will change with BER and it is difficult to be predicted. Since ETX does not reflect the change of BER in time with its long-term routing metric, we propose an opportunistic routing mechanism which combines with long-term and short-term routing metrics to solve the problem of ETX when it is applied in practice.

### 4.2. Opportunistic Routing Mechanism Combined with Short-Term Metric and ETX

The value of ETX of one link is the expected value of statistical link's quality during the latest 10 times. But if the quality of link fluctuates wildly during the period of 10 seconds, the predicted value will be in much warp and make the efficiency of routing lower. From the viewpoint of MAC layer, the node will choose the next hop which is able to reach the destination node and meet the routing algorithm. In the practical process of routing, choosing the path fixedly with minimum cost is not necessarily the best routing and the research in [23] also verified this view. In WMN, which adopts omnidirection antenna, the packets transmitted by source node could cover multineighbor nodes. This kind of routing mechanism with fixed path will not make full use of the sharing medium in wireless networks. So, based on the long-term routing metric such as ETX, combining with the state of links in short-term routing metric, we need to adopt a short-term routing metric to further restrict the routing. In the mechanism with short-term routing metric, the probability of link quality's change is considered as lower in a short period of time (millisecond scale). This mechanism probes the RTT of each candidate link every short period of time and determines the quality of links according to RTT. In this mechanism, some candidate links are selected according to ETX and the next hop will be chosen from these candidate links according to RTT. The short-term routing metric RTT is measured among the candidate links and nodes only maintain the latest one or several values of links' RTTs. Through enlarging the scale of routing in ETX, this mechanism increases the opportunity of being a choice of the links which has smaller ETX and better quality. At the same time, this mechanism increases the opportunity of data transmission over the links with higher quality under the assurance of QoS. And this routing mechanism is a kind of opportunistic method.

This opportunistic mechanism which takes long-term and short-term routing metrics into consideration uses heuristic method, and it is thought that the change of one link's state is continuous in a short period of time (tens of milliseconds). The current state of link is related to the previous two measurements and it will not change suddenly in most cases. This mechanism limits the additional short-term routing metric based on the global routing metric of long term and it improves the performance of routing under the assurance of QoS. Also, this mechanism is a method which takes both limitation of successful transmission count and performance of transmission into account through the additional storage, detection, and comparison in mesh routers.

### 4.3. Implementation of Opportunistic Routing Mechanism

The opportunistic routing mechanism based on ETX is a method which selects the path with suboptimum value of ETX. There exist multipaths which satisfy the QoS requirement and they will improve the performance of network through the routing of these candidate links by the constraint conditions of network contributes (available bandwidth, etc.). Combined with short-term routing metric and ETX, the opportunistic routing mechanism assures the choice of link with higher performance every time, while it does not guarantee the same links which are chosen. So the estimation of RTT and constraints of routing are needed in our routing mechanism.

It is thought that the change of one link's state is continuous in a short period of time and the threshold of ETX, ETX_threshold_, is set by network administrator or users. So the implementation of routing with constraints by the short-term routing metric of RTT shows as follows:

- the mechanism of short-term routing metric is set by mesh routers and they detect the links whose value of ETX is smaller than ETX_threshold_;
- the mesh routers send probe packets over these candidate links and acquire the current state of network according to RTTs;
- if there is an echo of ACK, the router records the RTT; otherwise, RTT is set by maximum value;
- for one of the candidate links, the router only maintains several historical values and estimates current state of network according to previous two records of RTT;
- the router chooses the link with the minimum RTT as the next hop.

**Figure 4:**
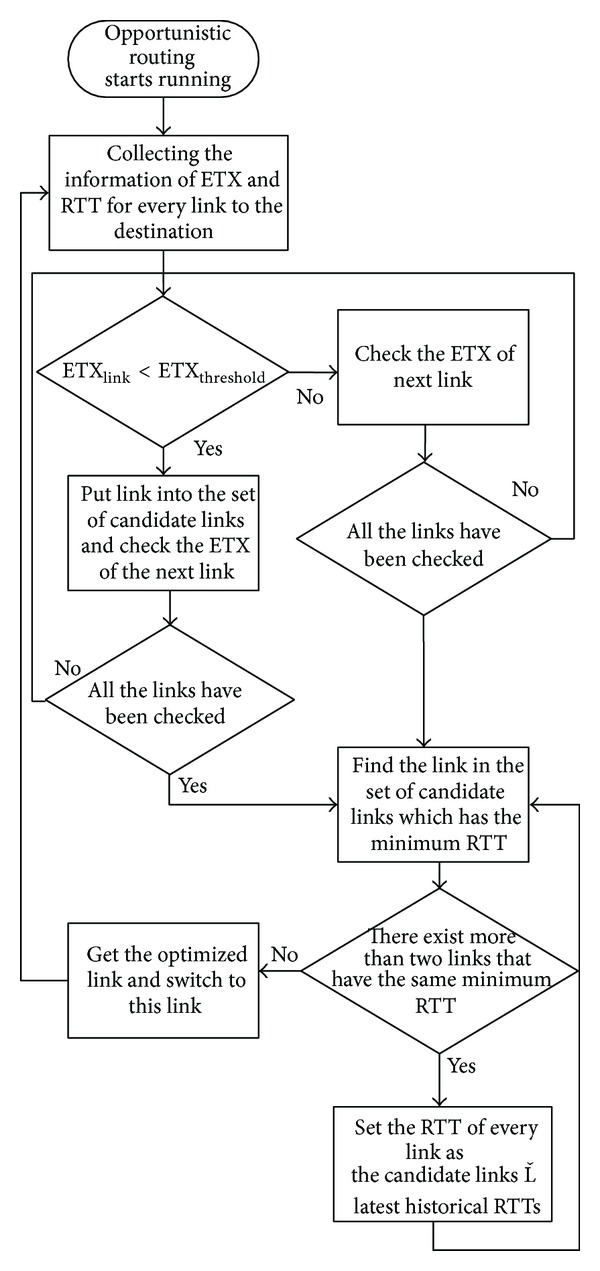
The process of opportunistic routing mechanism.

If the change of one link's state is continuous, the RTT of the next time can be estimated according to the empirical value of RTT: the RTTs of the latest two moments *T*_*n*−1_ and *T*_*n*−2_ are RTT_*n*−1_and RTT_*n*−2_, respectively, so RTT_*n*_ at *T*_*n*_ is considered RTT_*n*−1_ approximately. If RTT_*n*−1_ of candidate links are the same, the mechanism will choose the link with the minimum RTT_*n*−2_; otherwise, one of these links will be chosen randomly. The algorithm of opportunistic routing mechanism is shown in Algorithm 1 and the process of opportunistic routing is shown in Figure 4.

**Figure 5:**
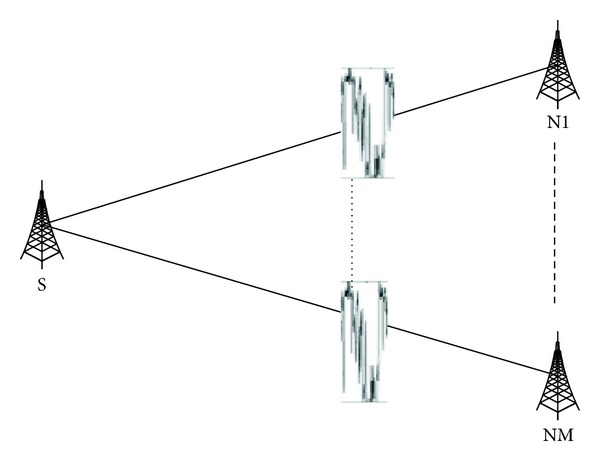
The routing of opportunistic mechanism.

In the scenario shown in Figure 5, the data source S records ETX and RTT of every link (Table 2) and ETX_threshold_ takes 4 hops. According to our opportunistic routing mechanism, node S will put links N2 and NM in the candidate set because their values of ETX are smaller than those of ETX_threshold_. Then node S probes the RTT of links N2 and NM and records the latest two results in the storage of S (the unit of RTT in Table 2 is millisecond). Since RTT_*n*−1_ of N2 and NM is the same, node S will choose NM as the next hop because of its smaller RTT_*n*−2_.

This mechanism which combines with long-term and short-term routing metric makes sure that not only ETX of selected link is under the assurance of QoS, but also the bandwidth of the link is the maximum among all of the candidate links. The measurement of long-term routing metric (ETX) is used as that in [2] which sends probe packets every 1 second, while mesh routers just send RTT probe broadcast packets periodically to the neighbor nodes which have the links in the candidate set. This mechanism avoids broadcast storm and will reduce the heavy burden of storage and processing in mesh routers.

## 5. Analyses of Opportunistic Routing Mechanism

The estimation of delay (Value_path_) from source to destination is expressed as the minimum one of the summation of RTT multiplied by EXT of each hop over every path:

(2) $$\begin{array}{c} {\text{Value}}_{\text{path}}=\min\left\{\underset{\text{hop}\;i}{\sum }({\text{ETX}}_{i}*{\text{RTT}}_{i})\;|\;\text{every}\;\text{path}\right\}. \end{array}$$

For the states of each mesh router over the path, we could transform the model of change of links' states to a model of Poisson distribution and it can be analyzed through Markov process. However, ETX and RTT of wireless links are ever-changing in practical applications, so it cannot be analyzed and implemented by the method of global modeling. On the other hand, there is no clear regulation of wireless link model and no preventative data of wireless condition could become the input for simulation. So we analyze the opportunistic routing mechanism from theory and implementation.

### 5.1. Analyses of Theory

The transmission rate of *i*th data packets of a link *l* at some moment is *R*_*i*_ = *α*∗(1/RTT_*i*_) and *α* is a factor of transmission time which is related to the size of probe packet. Suppose ETX is measured as *E*_*i*_ and *R*_*i*_ is independent of *E*_*i*_. Over the link layer, data packets are transmitted with the size of *b* and probe packets' size is also *b*. When the transmission rates of data packets and probe packets are the same, the expected transmission time of a packet with the size of *b* is *t*_*i*_ = (*b*/*R*_*i*_)∗*E*_*i*_ = RTT_*i*_ · *E*_*i*_. For the packet with the size of* B*, the time of transmitting this packet is shown in (3) ignoring the delay of router's scheduling:

(3) $$\begin{array}{c} T_{1}=\overset{i+\lceil B/b\rceil }{\underset{j=i}{\sum }}t_{j}=\overset{i+\lceil B/b\rceil }{\underset{j=i}{\sum }}{\text{RTT}}_{j}*E_{j}. \end{array}$$

For every* j*, RTT_*j*_ and *E*_*j*_ may be different. *E*_*j*_ is the long-term routing metric which will be modified every 1 second and RTT_*j*_ reflects the value of short-term routing metric. Suppose that the interval of RTT_*j*_'s change is *τ*, so RTT_*j*_ will change *n* = ⌊1/*τ*⌋ times during every* E*_*j*_'s update. If *τ* takes 15∗10^−3^ s and probe interval of ETX is 1 second, *E*_*j*_ will be updated after RTT_*j*_ changes 66 times. In this case, our opportunistic mechanism is able to detect the change of wireless links more accurately and make sure that every packet will be transmitted over the links with better performance.

It will spend ⌈*B*/*b*⌉ times for a packet with the size of *B* over the link with minimum value of ETX. Suppose the change of links is random and the maximum transmission rate of links is* R*, so the transmission rate of each packet is *ε*_*i*_ *R* (*ε*_*i*_ ∈ [0, 1]) and the minimum ETX of this link is *E*_min⁡_. The time of transmission is shown as follows:

(4) $$\begin{array}{c} T_{2}=\left(\overset{\left\lceil B/b\right\rceil }{\underset{i=1}{\sum }}\frac{b}{\varepsilon_{i}R}\right)*E_{\min}=\left(\overset{\left\lceil B/b\right\rceil }{\underset{i=1}{\sum }}\frac{1}{\varepsilon_{i}}\right){\text{RTT}}_{\min}*E_{\min}. \end{array}$$

Compared with (3) and (4), *E*_*j*_ is close to *E*_min⁡_ because *E*_*j*_ is limited by ETX_threshold_. So the margin of deviation between *E*_*j*_ and *E*_min⁡_ is very limited (*ξ*_*j*_ = *E*_*j*_/*E*_min⁡_). The value of RTT_*j*_ is the minimum one of all the candidate links and it is most close to RTT_min⁡_  (*κ*_*j*_ = RTT_*j*_/RTT_min⁡_). From (3) and (4), we can get the derivation of

(5) $$\begin{array}{c} \frac{T_{1}}{T_{2}}=\frac{\sum_{j=1}^{\left\lceil B/b\right\rceil }\kappa_{j}\xi_{j}}{\sum_{i=1}^{\left\lceil B/b\right\rceil }1/\varepsilon_{i}}. \end{array}$$

In special case, the critical value of *T*_1_/*T*_2_ is 1 when *B* is equal to *b* and *κ*_*j*_ · *ξ*_*j*_ = 1/*ε*_*i*_ which can also be expressed by

(6) $$\begin{array}{c} \varepsilon_{i}=\frac{{\text{RTT}}_{\min}}{{\text{RTT}}_{j}}*\frac{E_{\min}}{E_{j}}. \end{array}$$

In common conditions of wireless networks, the state of network accords with the uniform distribution from 0 to 1. So the expectation of the left of (6) is 0.5. The bigger the value of ETX_threshold_ is, the more links will be taken into the candidate set and RTT_*j*_ will be closer to RTT_min⁡_; however, *E*_min⁡_/*E*_*i*_ will be smaller at the same time. So there will also be more links taken in the candidate set with ETX_threshold_ of (*E*_min⁡_ + 1) when there are more links to the next hop.

At the same time, the actual and maximum throughput of l are Th_1_ and Th_2_, respectively, and their ratio is shown in (7) under the assumption that *B* is equal to *b*.

If the value of ETX_threshold_ decreases, the number of candidate links in set will also decrease. And then *E*_*j*_ will be closer to *E*_min⁡_; however, it will be unable to predict the changing of RTT_*j*_ because of the decrement of candidate links. At the same time, although sending RTT probe packet more frequently will make RTT_*j*_ more accurate, overmuch probe packets will lead to more collisions between packets and then *E*_*j*_ may be larger. So the settings of RTT probe packets and ETX_threshold_ are very significant to increase throughput of network. Under the assumption that ETX_threshold_ and the interval of RTT probe packets are optimized, since the value of *ε*_*i*_ is relatively constant, the ratio between Th_1_ and Th_2_ will be closed to 1. And the throughput of the chosen link will achieve the maximum. In particular, in relative dense network, this mechanism of routing which takes short-term and long-term routing metric into account will perform better:

(7) $$\begin{array}{c} \frac{{\text{Th}}_{1}}{{\text{Th}}_{2}}=\frac{B/\left(\sum_{j=1}^{\left\lceil B/b\right\rceil }\kappa_{j}\xi_{j}\right)}{B/\left(\sum_{i=1}^{\left\lceil B/b\right\rceil }1/\varepsilon_{i}\right)}=\frac{{\text{RTT}}_{\min}E_{\min}}{\varepsilon_{i}{\text{RTT}}_{j}E_{j}}. \end{array}$$

### 5.2. Performance Evaluation of Opportunistic Routing

**Figure 6:**
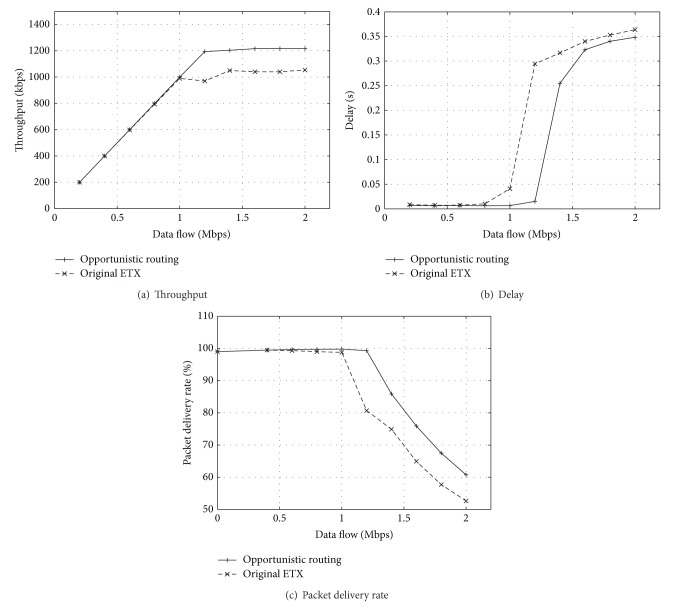
Performance comparison with different data rates.

We implement the function of opportunistic routing mechanism in NS-2.34 simulator, and then we process another simulation to evaluate the routing performance of opportunistic routing. In simulation, we also used the simple topology in Figure 1. The simulation period is 50 seconds and we vary the data rate of source-destination pair between* n*0 and* n*4 which make the data rate change from 0.2 Mbps to 2.0 Mbps. Then, the source node sends the packets to the destination node using original ETX and opportunistic routing. The results of performance comparison between original ETX and vp-ETX different data rates are shown in Figure 6.

Figure 6(a) shows that the throughput of opportunistic routing is always higher than original ETX when the data rate is higher than 1 Mbps and reaches the highest throughput which is about 1.216 Mbps. And the throughput is increased about 20% when the data rate is higher than 1 Mbps. The throughputs of original ETX and opportunistic routing are almost the same when the data rate is lower than 1 Mbps. However, the delay of opportunistic routing is a little lower than original ETX which could be seen in Figure 6(b). And the advantage of delay of opportunistic routing is more obvious when the data rate is higher. So the real time of opportunistic routing is better when network is dense and data rate is higher. To the packet delivery rate, opportunistic routing always performs better than original ETX at every data rate which could be seen in Figure 6(c).

From the analysis of the results of Figure 6, we find that opportunistic routing could perform better when the data rate of WMN is higher. So opportunistic routing mechanism is extremely suitable for the scenario of WMN with heavy data traffic.

### 5.3. Analyses of Implementation

Mesh routers need to support this opportunistic routing mechanism which combines with short-term routing metric for its implementation. Routers need to support the metric of ETX and detect RTT for a group of specific links. Particularly, when it detects that there is only one link whose ETX is smaller than the threshold, the mesh router should ignore the mechanism of short-term routing metric. On the other hand, our routing mechanism should be compatible with traditional mechanism with the metric of ETX and it will not enable the mechanism of short-term routing metric when ETX_threshold_ takes 0. For the choice of ETX_threshold_, the value of this threshold should be set according to the density of network. While the opportunistic mechanism cannot perform well in an extreme dense traffic network. The opportunistic mechanism avoids the bottleneck of transmission because of its avoidance of all packets transmitted over the same link simultaneously. Since the value of ETX changes at any time, the paths of packets transmitted may be different when the topology and ETX of network update, while the paths will not change much but will be fixed relatively when WMN adopts the opportunistic routing mechanism in some cases.

In practice, the coverage of IEEE802.11b is 300~500 meters and the speed of electromagnetic wave propagation is 2∗10^8^ meters per second. Although, considering the processing delay, RTT of adjacent neighbor nodes is far less than 1 millisecond, for WMN based on IEEE 802.16a (WiMAX) [24], the coverage of signal could be tens of kilometers. So RTT between neighbor nodes will reach the level of millisecond. Considering the influence with mesh routers' performance and compatibility with WiMAX of frequent detection, the level of short-term routing metric's probe interval should be millisecond [22].

Mesh routers will detect RTT of each link in the candidate set every 10 ms (empirical value), so it brings extra messages to network and requirement of routers' storage and computation ability. In the process of detecting RTT to neighbor nodes, the mesh router of detecting will broadcast probe packets and the links in the candidate set will respond, and then the router calculates RTT according to the response packet. In the process of probing and responding, the size of packet should be as small as possible; because of the smaller size of probe packet and longer probe interval, it does not bring too many messages to network. Since RTT is calculated by sender routers according to the current time and timestamp of response packet and candidate links are relatively limited, it will not bring too many mesh router operations and the performance of network transmission will not be influenced too much.

The opportunistic mechanism works across network and MAC layers. So it is responsible for routing and forwarding but not for order-preserving and error control of packets. The paths of packets transmitted may be different and packets arrive out of order if it adopts the opportunistic mechanism. For TCP flows, reliability of transmission can be assured through sequence number and congestion control mechanism, while, for UPD flows, it needs the process of order-preserving by application layer and it is the requirement of the opportunistic mechanism to upper layer protocols. It implies that the quality of link changes much when the packets of one date flow transmitted over different paths and the performance will decrease and even lead to the interruption of date flow if it still adopts original routing, while the opportunistic mechanism implements the transmission with higher efficiency and assures the quality of transmission through adjusting the routing of single packet in data flow. Moreover, this kind of mechanism avoids the situation that many data flows are transmitted over the same path and will not lead to failed transmission which is caused by the bottleneck of wireless links.

## 6. Conclusion

An opportunistic routing mechanism based on the routing metric of QoS-aware (ETX) is proposed in this paper. Through the simulation in this paper, ETX has proved that this kind of routing metric cannot reflect the quality of links quickly. However, our routing mechanism which combines with the routing metrics of short-term and long-term is proved theoretically to be able to overcome the weakness of ETX. Under the assurance of data flows' QoS, the link with highest performance will be chosen to transmit packets and this mechanism can better satisfy the requirement of wireless multihop network than the mechanism with ETX. This method also will change routing according to the quality of wireless links. Through analyses of performance and implementation, the opportunistic routing mechanism can increase the transmission efficiency of wireless links and improve the performance of wireless multihop network. In particular, in the scenario of heavy data traffic in WMN, opportunistic routing mechanism performs far better than original ETX mechanism.

We will find optimum values of ETX_threshold_ and probe interval of RTT in the future research. The further simulation of the topology of dense network is needed to verify the advantages of our routing mechanism. Also to prove the efficiency of the proposed mechanism, a verification of test-bed in a real scenario will be our future work.

## Figures and Tables

**Algorithm 1:**
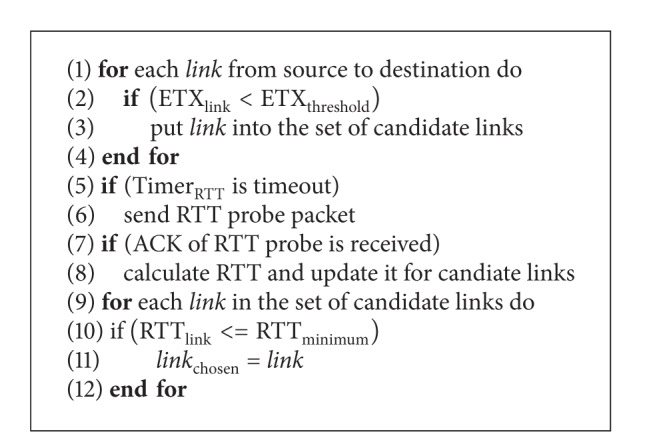
The algorithm of opportunistic routing mechanism.

**Table 1:** Parameter setting.

Parameter	Value
CBR/UPD ∗ 3	Variable
Packet size	1 Kbytes
Routing algorithm	OLSR_ETX
ETX probe interval	Minimum RTT in candidate set
Queue length	50
Mac type	802.11b
Bandwidth	11 Mbps
Propagation	Model two-ray ground
Antenna	Type Omniantenna
Frequency band	2.4 GHz
Simulation time	50 s
RTS/CTS	Off

**Table 2:** Parameter setting records in nodes.

Next hop	ETX	RTT_*n*−2_	RTT_*n*−1_
N1	5	0.4	0.4
N2	3	0.4	0.3
⋮	⋮		
NM	3	0.2	0.3

## Notations

*T*_*i*_: Transmission time for network packets
*E*_*i*_: ETX of link* i*
RTT_*i*_: Round-trip time of link* i*
*E*_min⁡_: Minimum ETX in candidate set
RTT_min⁡_: Minimum RTT in candidate set
ETX_threshold_: A threshold to filter candidate links
*B*: Size of data packets
*b*: Size of data payload over the wireless link
*R*_*i*_: Transmission rate of packets
Th_*i*_: Throughput of data packets over link* i.*
